# Supplementary figures and images for: DeepMIB: User-friendly and open-source software for training of deep learning network for biological image segmentation
Source: PLoS Comput Biol. 2021 Mar 2;17(3):e1008374. doi: 10.1371/journal.pcbi.1008374 (PMC7954287; doi:10.1371/journal.pcbi.1008374)

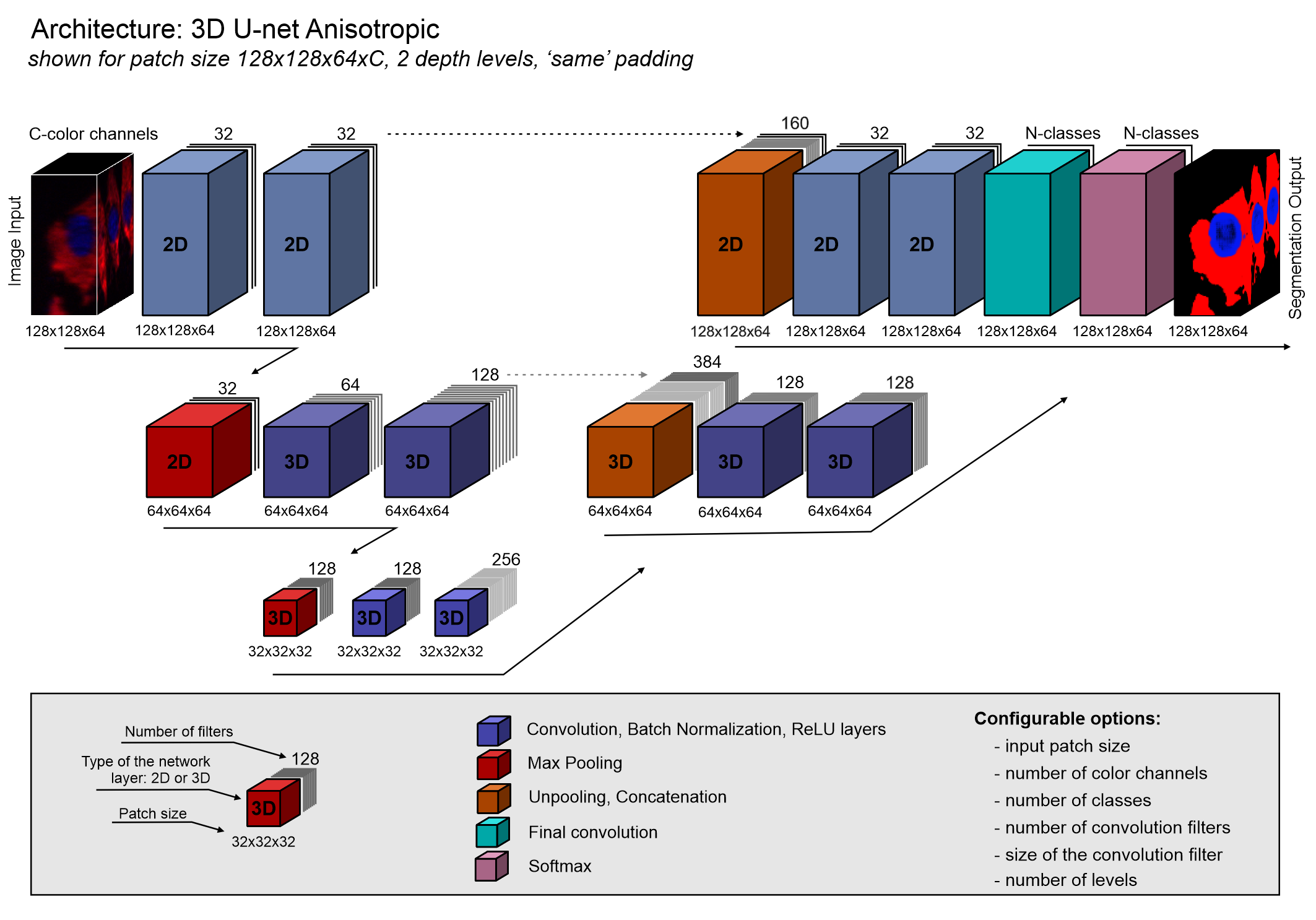

Supplement: S1 Fig — The architecture is based on a standard 3D U-net, where the 3D convolutions and the Max Pooling layer of the 1st encoding level are replaced with the corresponding 2D operations (marked using “2D” label). To compensate, the similar swap is done for the last level of the decoding pathway. The scheme shows one of possible cases with patch size of 128x128x64x2 (height x width x depth x color channels), 2 depth levels, 32 first level filters and using “same” padding. This network architecture can be tweaked by modifying configurable parameters and it works best for anisotropic voxels with 1 x 1 x 2 (x, y, z) aspect ratio. (TIF) [file pcbi.1008374.s001.tif]
